# Childhood Overweight/Obesity and Pediatric Asthma: The Role of Parental Perception of Child Weight Status

**DOI:** 10.3390/nu5093713

**Published:** 2013-09-23

**Authors:** Salma M. A. Musaad, Katie N. Paige, Margarita Teran-Garcia, Sharon M. Donovan, Barbara H. Fiese, the STRONG Kids Research Team

**Affiliations:** 1 Family Resiliency Center, Department of Human and Community Development, University of Illinois at Urbana Champaign, 904 W. Nevada, MC-081, Urbana, IL 61801, USA; E-Mail: smusaad@illinois.edu; 2 Division of Nutritional Sciences, University of Illinois at Urbana-Champaign, Urbana, IL 61801, USA; E-Mails: kpaige2@illinois.edu (K.N.P.); teranmd@illinois.edu (M.T.-G.); sdonovan@illinois.edu (S.M.D.); 3 Department of Food Science and Human Nutrition, University of Illinois at Urbana-Champaign, Urbana, IL 61801, USA; 4 The STRONG Kids Team includes Kristen Harrison, Kelly Bost, Brent McBride, Sharon Donovan, Diana Grigsby-Toussaint, Juhee Kim, Janet Liechty, Angela Wiley, Margarita Teran-Garcia and Barbara Fiese

**Keywords:** childhood obesity, pediatric asthma, food allergy, parental perception

## Abstract

Childhood obesity and asthma are on the rise in the U.S. Clinical and epidemiological data suggest a link between the two, in which overweight and obese children are at higher risk for asthma. Prevention of childhood obesity is preferred over treatment, however, in order to be receptive to messages, parents must perceive that their child is overweight. Many parents do not accurately assess their child’s weight status. Herein, the relation between parental perceptions of child weight status, observed body mass index (BMI) percentiles, and a measure of child feeding practices were explored in the context of asthma, food allergy, or both. Out of the children with asthma or food allergy that were classified as overweight/obese by BMI percentiles, 93% were not perceived as overweight/obese by the parent. Mean scores for concern about child weight were higher in children with both asthma and food allergy than either condition alone, yet there were no significant differences among the groups in terms of pressure to eat and restrictive feeding practices. In summary, parents of children with asthma or food allergy were less likely to recognize their child’s overweight/obese status and their feeding practices did not differ from those without asthma and food allergy.

## 1. Introduction

Childhood obesity is estimated at 17% in the United States [1]. The increase in childhood obesity has been linked to the increasing prevalence of related comorbidities, such as type 2 diabetes and asthma. The number of diagnosed cases of childhood asthma (<18 years) has increased from 8.7%, in 2001, to 9.6%, in 2009, tracking along with the growing prevalence of childhood obesity [1,2]. This upward trend is observed in all age groups. In preschool children (<4 years) asthma prevalence has been estimated to be 6.2%, with boys showing a consistently higher incidence than girls [3]. Although asthma and obesity have followed similar trends, studies have also shown that obesity in early-life may increase asthma risk. In the National Survey of Children’s Health, asthma in preschoolers (age 0–6) was 3% higher when the child was obese than normal weight [4]. While the underlying biological mechanisms linking obesity and asthma are not entirely clear, an excess of adiposity has been associated with a higher risk of asthma and more severe symptoms in both adults and children [5,6]. Further evidence of a relationship between asthma and obesity is demonstrated by the improvement of asthmatic symptoms and control in adults and children after significant weight loss or bariatric surgery [7,8]. As maintaining a healthy weight plays a critical role in reducing comorbidities in children, it is important that families are able detect when a child is overweight or at-risk for becoming obese.

Additionally, parents play a central role in determining how to feed their young children, and often make decisions based on whether they think their child is hungry or in need of more food [9]. Therefore, if a parent misperceives their child’s need for food, or weight status, and feeds them according to those misperceptions, their child may be at increased risk for obesity. Unfortunately, many parents fail to accurately assess their child’s weight. Parents of preschool-aged children are especially poor at approximating their child’s weight category [10] and 86% describe their child as normal weight when they are actually overweight as evidenced by elevated BMI [11]. More troubling is that few studies directly assess parental perception of child weight status, and even fewer studies explore this in the context of asthma. For example, one study examined parental report of weight and height and found it to be less sensitive and less specific for parents of children with asthma than parents of children without asthma [12]. Beyond the influence of child’s age and asthma status, other factors that may influence the accuracy of parental weight perceptions include parental weight status, maternal education, ethnicity, and child’s gender [11,13,14]. Regardless of the reason for parental misperception of child weight status, failing to recognize unhealthful behaviors may lead to worse outcomes and a more difficult implementation of treatment in the future. 

Though weight loss has been linked to improvements in asthma symptoms, dietary manipulations, including increased antioxidant and vitamin D intake and lower omega-6:omega-3 ratio, may also be beneficial [15]. However, evidence-based research in this area is still limited and even less well understood is how atopic conditions affect parental feeding practices. Dietary manipulations may occur by using controlling feeding practices such as restrictive feeding, pressure to eat, and monitoring. Research findings on the relationship between controlling feeding practices and child weight are inconsistent. Some studies suggest that such feeding practices are linked with an obese phenotype in later life by overruling a child’s ability to respond to internal signals of hunger and satiety [16]. Specifically, parents of obese children are more likely to use restrictive feeding practices, whereas parents of underweight children report pressuring their child to eat [17]. What is yet to be understood is the extent of how parents of children with asthma and/or food allergy apply such feeding practices and if their feeding practices differ by health condition. It seems reasonable that parents of children that are perceived to have food allergies will likely translate their concern by restricting their child’s feeding. Such restriction may be directed towards limiting the child’s food intake and variety. However, it may carry negative consequences by promoting an unbalanced or unhealthy diet, or by compromising the nutritional well-being of the child, potentially impacting weight gain and disease management. Whether they also experience pressure to eat or increased parental monitoring is unclear. The impact of asthma diagnosis on controlling feeding practices is also not well understood.

Another issue that complicates the food environment of children with asthma is the increased risk for food allergies [18]. The prevalence of food allergy among children <18 years of age significantly increased by 18% between 1997 and 2007 [19]. Children with asthma are likely to develop other allergic conditions including food allergy, and food allergy is associated with more severe asthma [20]. Children in the STRONG (Synergistic Theory and Research on Obesity and Nutrition Group) Kids cohort were 3.3-times more likely to have asthma, if they also have a food allergy [21,22]. The presence of food allergy is one indication that a child has allergic rather than nonallergic asthma. Allergic asthma is characterized by an increase in serum IgE in response to food allergens or other environmental stimuli. Individuals with allergic asthma also have important clinical and genetic differences when compared to those with nonallergic asthma [23]. Notably, adults and children with nonallergic asthma have shown a higher risk for obesity [24,25]. The mechanism for this increased obesity risk is not well understood and evidence is lacking as to whether or not environmental factors such as parental feeding practices play a role. Despite the recognized early origins of childhood obesity, data linking parental perception of child weight status with childhood asthma and food allergies, and the association with parental feeding practices in preschool-aged children is lacking.

## 2. Study Aims

As part of the secondary data analysis of the STRONG Kids data, this study aims to describe parental feeding practices and perceived body weight status of children as well as to investigate associated weight trends of children with asthma and food allergy based on measured BMI percentile. This study adds to the understanding of the relationship between obesity and asthma and the potential role that parental feeding behaviors in preschool-aged children may play for children with asthma and food allergies. Current programs and policies focus on prevention as the preferred action for reducing child obesity, however, preventative measures must begin early in life in order to be effective [26]. By exploring the parental misperception of weight status in preschoolers, our long-term goal is to encourage conversations between the patient and the health care team that inform rather than blame parents and increase opportunities for healthy behaviors to be established early in childhood.

## 3. Methods

Families of children between 2 and 5 years old were recruited as part of the STRONG Kids cohort, a 3-wave study conducted over 5 years that explores childhood obesity within a developmental ecological framework [21]. To ensure socio-economic and racial/ethnic diversity, an unequal probability sampling frame was used to identify licensed day care centers (*n* = 33) across five counties in East-central Illinois. Beginning in January 2009, 91% (*n* = 30) of the centers permitted recruitment of children and their parents. Written informed consent was obtained from the parents of the children involved in this study. Assent was obtained from the children to collect height and weight. This research was approved by the Institutional Review Board at the University of Illinois at Urbana-Champaign, and meets all requirements for ethical conduct for research with human subjects.

Response rates among parents ranged from 60% to 95% across centers. Of the 497 surveys collected for wave 1 at the time of this analysis, only children with measured height and weight (*n* = 407) were included. There were no significant differences in race/ethnicity, age, and gender of the child, or annual household income between children without a height or weight measurement and those with a height and weight measurement.

### 3.1. Data Collection

A comprehensive self-report questionnaire designed to collect data on demographic characteristics, feeding practices, food allergy, dietary and physical activity behaviors, and various aspects of parent-child relationships that moderate behaviors related to obesity risk among children was completed by the parents (biological or non-biological parent/other caregiver) of the enrolled children [21]. Parents completed surveys online or were mailed surveys, if they did not have Internet access. Height (cm) and weight (kg) of the children were collected at their child-care sites by trained research assistants. Height and weight was collected 3 times using a stadiometer (Seca, Model 242, Hanover, MD, USA) and scale (HealthOmeter, Model 349KLX, Jarden Consumer Solutions, Boca Raton, FL, USA), respectively, and the average was recorded across the three measurements.

#### 3.1.1. Demographics

Information on parent’s gender, marital status, parent’s race/ethnicity, education, annual household income, child’s gender, child’s race/ethnicity, and health care coverage was collected from the parent.

Prenatal history, any chronic medical conditions in the child, and family history in the biological relatives of the child were collected. Child food allergies or sensitivities were collected using questions adapted from a validated questionnaire [27]. Food allergy was determined based on positive parental report of food allergy or sensitivity to cow milk, peanuts, other nuts, egg, soy, fish, wheat, sesame, artificial sweeteners, shellfish, fruits, vegetables, and other foods. A child was considered to have a food allergy if he/she was allergic to at least one type of food, and no food allergy if he/she was not allergic to any food. Asthma was determined based on responses to the question “Has your child been diagnosed with any chronic medical conditions (e.g., PKU, type I diabetes, cystic fibrosis, asthma, inborn error of metabolism)?” Child BMI was calculated as weight (kg)/height^2^ (m^2^) and was converted into age and gender specific BMI percentiles [28].

#### 3.1.2. Parental Feeding Practices and Perceived Weight

The STRONG Kids panel survey included the Child Feeding Questionnaire (CFQ) items [29]. The CFQ is a commonly used self-report of parental beliefs, attitudes, and practices regarding child feeding that includes 28 items presented on a 5 point Likert-scale. The CFQ items are used to compose 7 factors including perceived responsibility (Cronbach’s alpha = 0.86), concerns about child weight (Cronbach’s alpha = 0.80), restriction (Cronbach’s alpha = 0.78), pressure to eat (Cronbach’s alpha = 0.74), monitoring (Cronbach’s alpha = 0.86), perceived parent weight (Cronbach’s alpha = 0.78), and perceived child weight (Cronbach’s alpha = 0.79). The CFQ assessed the child’s and parents’ weight status history by obtaining their weight status at different time periods. According to the CFQ, perceived child weight was assessed by taking the mean of 3 items that address the parental perception of the child’s weight when the child was 0–11 months, 12–23 months, and currently. Perceived parent weight was assessed by taking the mean of 4 items that address the parental self-perception of weight status when the parent was 5–10 years old, 11–19 years old, in their 20s, and currently. All perceived weight status items were measured on a scale of markedly underweight, underweight, average, overweight, or markedly overweight. Only the currently perceived child weight was used in correlation analyses (see Statistical Analysis section).

### 3.2. Statistical Analysis

The child’s asthma and food allergy status was categorized into 1 of 4 groups: no asthma or food allergy, food allergy only, asthma only, and asthma and food allergy. A food allergy index (1–3) was created by categorizing the number of food allergies (1–2, 3–4, 5, or more) to examine the effect of an incremental increase in the count of food allergy. The BMI percentiles were initially categorized into 4 categories of underweight (<5th percentile), healthy weight (5th to <85th percentile), overweight (85th to <95th percentile), and obese (≥95th percentile). Due to the limited sample size of underweight (*n* = 10) and obese (*n* = 31) children, participants were classified into 2 groups of either healthy weight, by combining the first 2 categories, or overweight/obese, by combining the third and fourth categories. Only 37 children were considered to be (reported as) markedly underweight and underweight as perceived by the parent. Thus, in order to increase the analytic sample size, children who were considered to be markedly underweight, underweight, and average by the parent were combined into 1 group of healthy weight children as perceived by the parent, and children who were considered overweight or markedly overweight by the parent were combined into another group of overweight/obese children as perceived by the parent. Similar results were observed after excluding children who were underweight according to BMI percentiles and children who were perceived to be markedly underweight or underweight by the parent (not shown). Therefore, results using the combined weight status categories are reported in this manuscript. The general association of the continuous child BMI percentiles and the parental perception of the child’s current weight status with the CFQ subscales were examined using the Spearman’s rank correlation coefficient (Spearman’s rho). The child’s current weight status was 1 of the 3 components of the perceived child weight subscale and addressed the parental perception of the child’s weight in the current time period only. Comparisons of categorical data were performed using the Chi square test or Fisher’s exact test. The Cochran-Armitage exact trend test was used to explore trends in the association of 2 categorical variables. CFQ subscales were compared across groups using the Analysis of Variance (ANOVA). The Wilcoxon 2-sample test with *t*-approximation or Kruskal-Wallis test was used to compare continuous data that was not normally distributed. A 2-tailed *p* value <0.05 was considered statistically significant. No adjustment for multiple testing was performed in this preliminary analysis. All analyses were conducted using SAS version 9.3 (SAS Institute, Cary, NC, USA). 

## 4. Results

### 4.1. Participants

The characteristics of the study population are presented in Table 1. Out of the 407 child participants, 51.8% were males, and 5.4% were Hispanic. Out of the non-Hispanic children, 26% were Black or African-American, 57.3% White, 10.1% Asian, and 1.2% American Indian or Hawaiian Native. A total of 94 children (23.1%) were classified as overweight/obese and 313 (76.9%) as healthy weight according to BMI percentiles. Median age was 42 months (25th percentile = 35, 75th percentile = 47) and it did not differ by BMI percentile category. The 332 children (81.6%) who did not have asthma or food allergy consisted of 253 healthy weight children and 79 overweight/obese children; the 38 (9.3%) who had food allergy only consisted of 31 healthy weight and 7 overweight/obese; the 30 (7.4%) who had asthma only consisted of 23 healthy weight and 7 overweight/obese; the 7 (1.7%) who had both asthma and food allergy consisted of 6 healthy weight and 1 overweight/obese. A total of 89% of the parents were female, 69% married or cohabitating, 23% single, 94% non-Hispanic, 13% completed high school, only 31% completed some years of college or technical school, and 56% completed college or post-graduate work. As shown in Table 1, only the race of the child, and parental education, and employment, differentiated the healthy weight from overweight/obese children. Black or African-American children of employed and less educated parents were more likely to be overweight/obese.

### 4.2. Prevalence of Child BMI Percentiles by Asthma-Food Allergy Status

Median BMI age and sex-specific percentiles differed by the asthma-food allergy status group, being 62.5 in children with no asthma or food allergy, 64.4 in those with food allergy only, 70.5 in those with asthma only, and 72.3 in children with asthma and food allergy. These differences were not significant. Additionally, among children with food allergy, the proportion of overweight/obese children was about twice that in children with five or more food allergies (33.3%) compared to children with one to two food allergies (17.1%), but this relationship did not achieve statistical significance.

**Table 1:** Characteristics of the total study sample (*n* = 407), shown as number (%) or median (25th percentile, 75th percentile).

Characteristic	Number (% of total sample)	Child’s BMI percentile		*p* Value
		Healthy weight (*n* = 313)	Overweight/obese (*n* = 94)	
*Parent’s gender*				0.344
Male	41 (10.1)	34 (10.9)	7 (7.5)	
Female	364 (89.4)	278 (89.1)	86 (92.5)	
Unknown/missing	2 (0.5)			
*Relationship of parent to child*				0.278
Biological parent	383 (94.1)	293 (94.2)	90 (97.8)	
Adoptive	10 (2.5)	10 (3.2)	0	
Relative	7 (1.7)	6 (1.9)	1 (1.1)	
Other	3 (0.7)	2 (0.6)	1 (1.1)	
Unknown/missing	4 (0.9)			
*Marital status*				0.137
Single	94 (23.1)	65 (21.0)	29 (31.9)	
Married/civil union/co-habitating	282 (69.3)	226 (73.1)	56 (61.5)	
Separated	7 (1.7)	5 (1.6)	2 (2.2)	
Divorced/widowed	17 (4.2)	13 (4.2)	4 (4.4)	
Unknown/missing	7 (1.7)			
*Highest level of schooling of parent*				0.007 *
Grade school/some high school/high school graduate	53 (13.0)	36 (11.5)	17 (18.3)	
Some college or technical school	126 (30.9)	89 (28.4)	37 (39.8)	
College graduate/Post graduate work	227 (55.8)	188 (60.1)	39 (41.9)	
Unknown/missing	1 (0.3)			
*Parent’s employment status*				0.042 *
Employed/self employed	270 (66.3)	200 (64.5)	70 (75.3)	
Student	74 (18.2)	59 (19.0)	15 (16.1)	
Unemployed	19 (4.7)	15 (4.8)	4 (4.3)	
Stay at home	35 (8.6)	33 (10.7)	2 (2.2)	
Retired/disabled	5 (1.2)	3 (0.9)	2 (2.2)	
Unknown/missing	4 (0.9)			
*Annual household income*				0.569
$24,999 or less	106 (26.0)	76 (24.8)	30 (32.6)	
$25,000 to $39,999	49 (12.0)	38 (12.4)	11 (11.9)	
$40,000 to $69,999	70 (17.2)	54 (17.7)	16 (17.4)	
$70,000 to $99,999	74 (18.2)	57 (18.6)	17 (18.5)	
$100,000 or more	75 (18.4)	63 (20.6)	12 (13.0)	
Unknown/missing	33 (8.1)			
*Healthcare coverage*				1.0
Yes	378 (92.9)	291 (97.3)	87 (97.8)	
No	10 (2.5)	8 (2.7)	2 (2.3)	
Unknown/missing	19 (4.7)			
*Child’s age (months)*	42 (35, 47)	42 (35, 47)	42 (35, 48)	0.561
*Child’s BMI percentile*	63.9 (39.1, 84.4)	54.3 (31.9, 71.2)	92.2 (89.4, 96.1)	<0.0001 **
*Child’s gender*				0.814
Male	211 (51.8)	161 (51.4)	50 (53.2)	
Female	196 (48.2)	152 (48.6)	44 (46.8)	
*Child’s race/ethnicity*				0.005 *
Hispanic	22 (5.4)	20 (6.4)	2 (2.1)
Black or African American	106 (26)	77 (24.6)	29 (30.9)	
White	233 (57.3)	174 (55.6)	59 (62.8)	
Asian	41 (10.1)	39 (12.5)	2 (2.1)	
American Indian or Hawaiian Native	5 (1.2)	3 (0.9)	2 (2.1)	
*Child asthma status*				0.823
No asthma	370 (90.9)	284 (90.7)	86 (91.5)	
Asthma	37 (9.1)	29 (9.3)	8 (8.5)	
*Child food allergy status*				0.369
No food allergy	362 (88.9)	276 (88.2)	86 (91.5)	
Food allergy	45 (11.1)	37 (11.8)	8 (8.5)	
*Child combined asthma-food allergy status*				0.834
No asthma or food allergy	332 (81.6)	253 (80.8)	79 (84.0)	
Food allergy only	38 (9.3)	31 (9.9)	7 (7.5)	
Asthma only	30 (7.4)	23 (7.4)	7 (7.5)	
Asthma and food allergy	7 (1.7)	6 (1.9)	1 (1.1)	
*Child number of food allergies*				0.782
1-2 food allergies	35 (8.6)	29 (78.4)	6 (75.0)	
3-4 food allergies	7 (1.7)	6 (16.2)	1 (12.5)	
5 or more food allergies	3 (0.7)	2 (5.4)	1 (12.5)	

Percentages may not always add up to 100 due to rounding. * Chi square or Fisher’s exact test comparing overweight/obese with healthy weight child BMI percentile categories. ** Wilcoxon two-sample test.

### 4.3. Discordance in the Classification of Overweight/Obesity

Table 2 shows the relationship between the BMI percentiles and the parental perception of the child’s current weight status. About eight times more children (93 *vs.* 12 children) were identified as overweight/obese according to BMI percentiles than by parental report. Significant discordance (*p* = 0.009) and very little agreement (kappa = 0.08, 95% confidence interval ((CI) = 0.01, 0.16) was noted between the child’s current weight status as perceived by the parent and measured BMI percentiles. Among the 93 children categorized as overweight/obese according to BMI percentiles, most (92.5%) were perceived to be healthy weight by the parent. In contrast, among 305 children considered to be healthy weight according to the BMI percentiles, 98.4% were perceived to be healthy weight by the parent. When stratified by gender, the association remained significant in boys, but not in girls (data not shown).

**Table 2:** Comparison of the number (%) of children by body mass index (BMI) percentile categories and the parental perception of the child’s current weight status.

Parent perception of child’s current weight status	Child’s BMI percentile		
	Healthy weight	Overweight/obese	*p* Value
Healthy weight	300 (98.4)	86 (92.5)	0.009 *
Overweight/obese	5 (1.6)	7 (7.5)	

Percentages are reported based on the child BMI percentile category. There were 9 children with missing information about perceived weight status from the parent. * Fisher’s exact test comparing child BMI percentiles with parental perception of the child’s current weight status.

Two discordant groups were identified. The first included 86 children who were classified as overweight/obese according to BMI percentiles, but not according to the parent. The second included five children who were classified as overweight/obese according to the parent, but not according to BMI percentiles. When comparing the prevalence of asthma-food allergy status across the discordant groups, children in the first discordant group (identified as overweight/obese by BMI percentiles, but not perceived as such by the parent) were found to have more children with asthma and food allergy compared to the second discordant group (children perceived as overweight/obese by the parent, but not identified as such by BMI percentiles). In the first discordant group, a total of 13 children had asthma only or food allergy only. In the second discordant group, only one had food allergy and none had only asthma. None of the discordant groups had children with both asthma and food allergy. 

**Figure 1:**
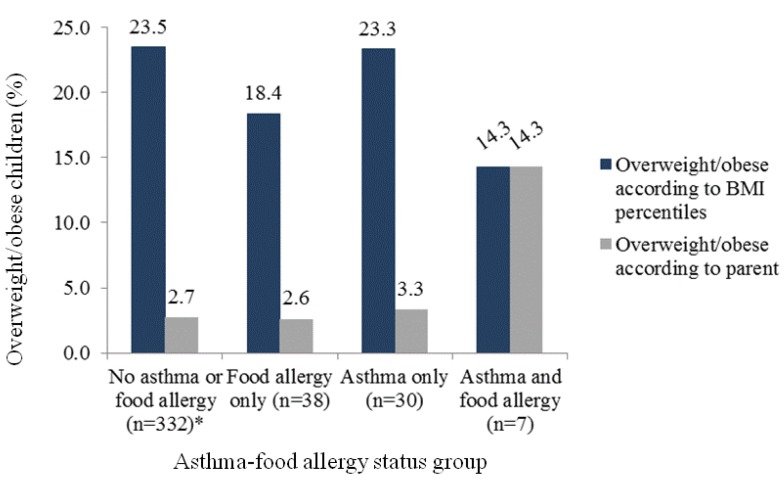
Overweight/obese children (%) in the asthma-food allergy status groups according to BMI percentiles (dark blue) and parental perception of the child’s current weight (gray).

The distribution of the child’s BMI percentiles and the parental perception of the child’s current weight status across asthma-food allergy status categories was examined. Figure 1 shows the proportion of overweight/obese children according to BMI percentiles and the parental perception of their child’s current weight status within each asthma-food allergy category. Except for children with both asthma and food allergy, the prevalence of obesity, when objectively measured using BMI percentiles, was consistently higher than parental perception. Further, a higher proportion of overweight/obese children among children with asthma only compared to children with food allergy only was observed irrespective of the obesity measure (23.3% *vs.* 18.4% according to measured BMI percentiles and 3.3% *vs.* 2.6% according to parental perception).

**Figure 2:**
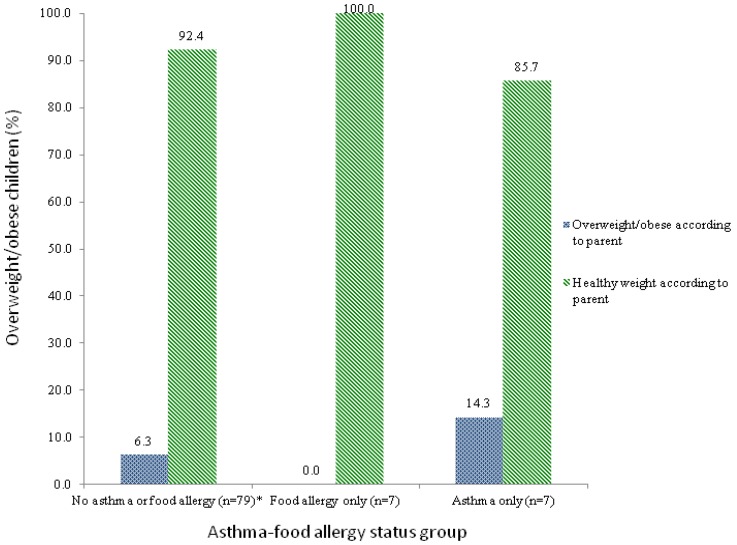
Child’s weight status (%) as perceived by the parent in children classified as overweight/obese according to BMI percentiles.

The discordance between the measured child’s BMI percentiles and the parental perception of the child’s current weight status was evaluated across three of the four levels of asthma-food allergy status (no asthma or food allergy, food allergy only, and asthma only). As there was only one child in the asthma and food allergy group who was classified as overweight/obese by both BMI percentiles and the parent, no definitive conclusions could be drawn about this group. Figure 2 shows the proportion of healthy weight and overweight/obese children according to parental perception of the child’s current weight status within children with no asthma or food allergy, food allergy only, and asthma only who have been classified as overweight/obese according to BMI percentiles. Only the comparison among children with no asthma or food allergy achieved statistical significance whereby five (6.3%) were perceived as such by the parent (*p* = 0.039) but the remaining 73 children (92.4%) were not. None of the seven children with food allergy only who were classified as overweight/obese according to BMI percentiles were perceived as such by the parent. In children with asthma only that were classified as overweight/obese by BMI percentiles, 6 (85.7%) were not perceived as such by the parent while only one (14.3%) was. Thus, the weight status of 13 (seven with food allergy only and six with asthma only) out of 14 (92.9%) children with asthma only or food allergy only was incorrectly perceived by the parent (discordant).

* *p* = 0.039 using Fisher’s exact test comparing child BMI percentile categories with child weight status as perceived by the parent among children with no asthma or food allergy.

* *p* = 0.039 using Fisher’s exact test comparing child BMI percentile categories with child weight status as perceived by the parent among children with no asthma or food allergy.

### 4.4. Feeding Practices across Asthma-Food Allergy Status Categories

Table 3 displays the mean score of the CFQ subscales in the asthma-food allergy status groups. Perceived child weight (*p* = 0.037) and concern about child weight (*p* = 0.022) differed significantly by asthma-food allergy status. Mean levels were higher in children with asthma compared to children with food allergy only.

**Table 3:** Mean (standard deviation) of Child Feeding Questionnaire (CFQ) subscale scores in the asthma-food allergy status groups.

CFQ subscale	Asthma-food allergy status								*p* Value *
	No asthma or food allergy		Food allergy only		Asthma only		Asthma and food allergy		
	*n*	Mean (SD)	*n*	Mean (SD)	*n*	Mean (SD)	*n*	Mean (SD)	
Perceived parent weight	321	3.16 (0.473)	38	3.17 (0.520)	28	3.26 (0.702)	7	3.21 (0.529)	0.352
Perceived child weight	321	2.92 (0.384)	38	2.77 (0.509)	29	3.01 (0.484)	7	2.95 (0.230)	0.037
Perceived responsibility	326	4.47 (0.691)	38	4.56 (0.635)	28	4.73 (0.567)	7	4.90 (0.252)	0.160
Concern about child weight	326	1.83 (1.019)	38	1.46 (0.741)	29	1.68 (0.843)	7	2.24 (1.499)	0.022
Restriction	317	2.89 (0.733)	35	2.82 (0.642)	29	2.77 (0.586)	6	2.75 (0.766)	0.319
Pressure to eat	321	2.41 (0.937)	38	2.29 (0.958)	29	2.65 (0.937)	7	2.71 (0.822)	0.286
Monitoring	325	4.09 (0.911)	38	4.17 (0.919)	29	4.32 (0.664)	7	3.76 (1.607)	0.741

* ANOVA comparing mean CFQ subscale scores across asthma-food allergy status categories.

Perceived child weight (Spearman’s rho = 0.367, *p* < 0.0001), perceived parent weight (Spearman’s rho = 0.225, *p* < 0.0001), concern about child weight (Spearman’s rho = 0.198, *p* < 0.0001), perceived responsibility (Spearman’s rho = 0.121, *p* = 0.015), and pressure to eat (Spearman’s rho = −0.159, *p* = 0.002) were weakly correlated with child BMI percentiles. Concern about child weight (Spearman’s rho = 0.137, *p* = 0.003), restriction (Spearman’s rho = 0.126, *p* = 0.007), pressure to eat (Spearman’s rho = −0.107, *p* = 0.019), and perceived child weight (Spearman’s rho = 0.616, *p* < 0.0001) were weakly to moderately correlated with the parental perception of the child’s current weight status. Table 4 presents the correlation of the CFQ subscales and child BMI percentiles in the asthma-food allergy status groups, and Table 5 presents the correlation of the CFQ subscales and parental perception of the child’s current weight status (a subcomponent of the perceived child weight subscale indicating current weight status only) in the asthma-food allergy status groups. Similar trends were seen with the exception of a markedly strengthened correlation between concern about child weight and BMI percentiles in children with asthma and food allergy (Spearman’s rho = 0.778, *p* = 0.039). Restrictive feeding practices were associated with parental perception of their child’s current weight only in children with no asthma or food allergy.

**Table 4:** Association of the CFQ subscales with child BMI percentiles using Spearman’s rank-order correlation.

CFQ subscale			Asthma-food allergy status					
	No asthma or food allergy	*p* Value	Food allergy only	*p* Value	Asthma only	*p* Value	Asthma and food allergy	*p* Value
	Correlation with child BMI percentiles							
Perceived parent weight	0.242	<0.0001	0.239	0.147	0.119	0.548	−0.408	0.364
Perceived child weight	0.363	<0.0001	0.434	0.007	0.409	0.028	0.079	0.865
Perceived responsibility	0.102	0.065	0.328	0.044	0.162	0.409	−0.612	0.144
Concern about child weight	0.178	0.001	0.265	0.108	0.188	0.329	0.778	0.039
Restriction	0.044	0.431	−0.217	0.210	−0.006	0.976	0.429	0.397
Pressure to eat	−0.178	0.001	−0.124	0.459	−0.071	0.713	−0.148	0.751
Monitoring	0.027	0.623	−0.039	0.818	−0.263	0.169	−0.491	0.263

**Table 5:** Association of the CFQ subscales with parental perception of the child’s current weight status using Spearman’s rank-order correlation.

CFQ subscale	Asthma-food allergy status							
	No asthma or food allergy	*p* Value	Food allergy only	*p* Value	Asthma only	*p* Value	Asthma and food allergy	*p* Value
	Correlation with parental perception of child’s current weight status							
Perceived parent weight	0.091	0.073	−0.028	0.857	0.077	0.68	−0.509	0.109
Perceived child weight	0.642	<0.0001	0.682	<0.0001	0.577	<0.001	−0.524	0.098
Perceived responsibility	0.060	0.235	−0.011	0.943	−0.058	0.755	−0.593	0.054
Concern about child weight	0.131	0.009	0.206	0.175	−0.150	0.413	0.507	0.111
Restriction	0.134	0.009	−0.062	0.697	0.306	0.094	0.058	0.873
Pressure to eat	−0.108	0.034	−0.238	0.116	0.044	0.811	−0.101	0.767
Monitoring	0.034	0.505	0.018	0.909	−0.105	0.568	−0.514	0.106

## 5. Discussion

We explored the relation between parental perception of child weight status and observed BMI percentiles among children with a median age of 3.5 years, and compared this relation between those with food allergy only, asthma only, and those with asthma and food allergy. Consistent with previous research, children with asthma were more likely to be overweight or obese [30,31]. We also noted that children with both asthma and food allergy had the highest median BMI percentiles. Due to the limited sample size, we were not able to detect reliable group differences in this small sample. Future research with larger samples should consider the combined risk of asthma and food allergy in childhood obesity.

Previous research shows that about 30%–80% of parents do not correctly identify their child’s weight status especially among parents of preschool-aged children [10,14,32,33,34]. We also found that the majority of parents did not perceive their children as overweight/obese, even when classified as overweight/obese by BMI percentiles. For those children who were classified as overweight/obese, less than 10% of the parents perceived their children to be as such. This discordance between BMI percentiles and parental perception persisted in children with asthma or food allergy. Out of the children with asthma or food allergy that were classified as overweight/obese by BMI percentiles, 93% were not perceived as overweight/obese by the parent. Parents who do not perceive their child as overweight are less likely to offer a healthy dietary lifestyle, which may have consequences for how the parents regulate their child’s food intake as well as their receptiveness to messages regarding prevention of excessive weight gain [29,35].

In the STRONG Kids cohort, an assessment of parental feeding perceptions, attitudes, and practices suggested that parents of children with asthma perceived them to be heavier than parents of children with food allergy. Furthermore, mean scores for concern about child weight were significantly higher in children with asthma and food allergy (2.24) than children with asthma only (1.68) and food allergy only (1.46). In fact, concern about child weight was associated with child BMI percentiles most strongly among children with asthma and food allergy. These findings suggest that the observed differences in parental concern about their child’s weight will translate into differences in specific feeding practices to control their child’s eating, including monitoring, restriction, and pressure to eat, and that these differences may be largest for children with both asthma and food allergy compared to the other groups. However, this study found that families of children with asthma and food allergy had the lowest monitoring scores. Furthermore, there were no significant differences among the groups in terms of pressure to eat and restrictive feeding practices. Additionally, restrictive feeding practices and concern about child weight were weakly yet significantly associated with parental perception of their child’s current weight in children with no asthma or food allergy. These data suggest that parents restrict food intake in response to their concern about what they perceive the child weight status to be, rather than the child’s measured BMI percentiles. It also suggests that parents of children with asthma or food allergy may not always be receptive to information from their pediatrician or health care team on appropriate feeding practices. Overall, similar trends were seen after excluding children who were considered to be markedly underweight or underweight according to the parental perception of child weight status and BMI percentiles. 

One of the major limitations of this study is the small number of children with asthma only, food allergy only, or both who were also classified as overweight/obese according to BMI percentiles. Therefore, this study may not be generalizable to the US population, although the prevalence of asthma and allergy in STRONG Kids is similar to a nationally representative aged-matched sample [18,22]. In addition, no conclusions about causality can be made due to the cross-sectional design. Results from this study may not be comparable to other studies due to differences in the weight status categories for both BMI percentiles and parental perception of child weight, in which underweight children were combined with healthy weight children and overweight children were combined with the obese. Further, parents of children with asthma and/or food allergies face several challenges when trying to manage their child’s weight. For example, the severity of the asthma, the side effects of asthma medications, or the child’s preference for certain foods can influence the parent’s feeding practices. It would be interesting to account for these factors when exploring parental feeding practices in future studies. The STRONG Kids survey did not address the question of asthma severity, therefore, it was not possible to relate asthma severity with the number of food allergies. Additionally, this study does not account for the extent of physical activity in the children, and does not have enough sample size to stratify the analyses by ethnicity. Lastly, a self-report questionnaire was used including the asthma report by the parent, thus subjecting the data to reporting bias. However, given the young age of this sample, we expect the reporting of asthma to be low, because asthma is difficult to diagnose before the age of five years [36]. Longitudinal data from a larger sample that has been collected to answer the study questions would help clarify the present findings, and shed light on which children who currently do not have asthma or food allergy are destined to develop one or more of these conditions. The strengths of this study are that children’s height and weight were objectively measured, the CFQ is a validated measure of child feeding perceptions, attitudes, and practices, and that this is a very young population of children where parental feeding practices play a major role in their likelihood for obesity.

## 6. Conclusions

In conclusion, a significant proportion of parents of overweight/obese preschoolers, including children with asthma, food allergy, or both, underestimated their child’s weight status. Parents of children with both asthma and food allergy showed the greatest concern for their child’s weight, and their concern was most strongly associated with child’s BMI percentiles in this group. This concern, however, did not appear to translate into differences in the use of restriction, monitoring, or pressure to eat between children with no asthma or food allergy and those with asthma, food allergy, or both. Therefore these parents may ignore health messages targeting obesity prevention, thus increasing their children’s likelihood for obesity.
